# Mental distress and perceived wealth, justice and freedom across eight countries: The invisible power of the macrosystem

**DOI:** 10.1371/journal.pone.0194642

**Published:** 2018-05-02

**Authors:** Saskia Scholten, Julia Velten, Jürgen Margraf

**Affiliations:** Department of Psychology, Ruhr-Universität Bochum, Bochum, Germany; University of Illinois at Urbana-Champaign, UNITED STATES

## Abstract

Health and well-being have been related to macro-level factors such as income, income inequality or socioeconomic status. With regard to the increasing burden of disease due to mental disorders worldwide, the association between the macrosystem and mental distress should be further explored, too. In this context, the subjective evaluation of the macrosystem might play an important role. In the present exploratory study, we assessed symptoms of depression, anxiety and stress as well as perceived wealth, justice and freedom in population-based surveys in Spain, France, Germany, Poland, Russia, Sweden, the United Kingdom and the United States of America (n ≈ 1000 per country). The Swedish sample presented the lowest symptom ratings of depression, anxiety and stress and the highest self-rated health. The results also indicated that the subjective evaluation of the macrosystem matters in respect to mental distress. The complete model, including the control variables country, gender, age and education, and perceived wealth, justice and freedom predicted depression, anxiety and stress symptoms explained 8% of the variance of each symptom cluster. The present results encourage research to consider the macrosystem, and the subjective evaluation of macro-level factors, as a relevant component in biopsychosocial models of mental distress.

## Introduction

A growing burden of disease due to mental disorders has put the improvement of mental health on the global agenda (Resolution WHA65.4, [1]). The WHO Mental Health Action Plan emphasizes social, cultural, economic, political and environmental factors besides individual attributes as determinants of mental health and disorders [2]. However, the focus of most research related to mental distress is set on the characteristics of the microsystem, such as the person itself and its biological vulnerability as well as stress, family or social support [3]. In order to understand the etiology of mental distress and to develop treatment strategies that exceed individual-centered rationales, taking the macrosystem into account is a valuable addition to current research.

The “power of the macrosystem” has mainly been studied in relation to mortality, morbidity, health and well-being (e.g., [4–7]). But an increasing number of studies support the WHO’s statement by providing evidence for the association between the macrosystem and mental distress [8–13]. Conceptually, this point of view has been taken as early as 1977 by Engel who introduced the biopsychosocial model to explain mental distress opposing the reductionist view of the biomedical model [14]. He stresses that social factors are crucial in the determination of distress (p. 132). His theoretical framework describes the human being as a system within the continuum of natural systems [15]. Bronfenbrenner [16] differentiates these mutually related systems into the microsystem (e.g., family or school), the mesosystem (describing the interconnection of different microsystems), the exosystem (e.g., neighbors or mass media) and the macrosystem (e.g., culture and society, including socioeconomic status, values). In present research, macro-level factors are mostly measured with objective indicators such as income, income inequality or socioeconomic status (SES). Research has not focused on the subjective evaluation of macro-level factors and its role in the relationship between the macrosystem and the individual. An exception are the subjective SES and relative personal deprivation [17, 18]. These studies show that subjective evaluation predicts levels of mental distress much better than comparable objective indicators [19, 20]. Based on these findings, additional macro-level factors besides SES should be assessed subjectively to enhance our understanding of the relation between the individual perception of macro-level factors and mental distress. Among the symptoms of mental distress, symptoms of depression and anxiety should be focused because they are the most prevalent mental disorders worldwide [21, 22].

The present study aims to explore the association between the macrosystem, perceived macro-level factors and mental distress in a cross-cultural framework including eight countries. To the best of our knowledge, this is the first study that assesses symptoms of depression, anxiety and stress as well as macro-level factors subjectively, simultaneously and comparably across countries.

### Countries as macrosystems

The macrosystem refers to consistencies in the form and content of lower-order systems such as micro-, meso- or exosystems that exist on the level of larger social entities [16]. Countries are social entities that can be viewed as macrosystems because their lower-order systems share common characteristics such as the welfare-system. Based on the welfare-system, Esping-Andersen [23] introduced an approach to classify countries. He introduced “Three worlds of welfare”: liberal (e.g., the USA or UK), conservative (e.g., Germany) and social-democratic (e.g., Sweden). For comparative studies, the three clusters proved to be a robust and useful classification [24]. Looking at the relation between the welfare-system and health, the welfare-system explains about 10% of the variance of self-rated health across 21 European countries. Higher perceived self-rated health was reported in liberal and social welfare systems [4]. An association between mental distress or prevalence rates of mental disorders and welfare systems has not been established yet.

### Subjective macro-level factors: Perceived wealth, justice and freedom

Within macrosystems, only subjective data can reveal the interpretation of objective life circumstances [25]. Thus, the individual perception and subsequently the subjective evaluation of macro-level factors should be explored (p. 6, [26]). For the present study, we focused on wealth, justice and freedom, because they are important macro-level factors that vary across welfare systems and they have previously been related to health and mental health. Yet, they have not been studied in combination from a subjective perspective until now. Investigating wealth, justice and freedom in combination allows to detangle their potentially differential effects on mental distress.

#### Wealth

The so-called “Easterlin paradox” was one of the first systematic descriptions of the relation between a macro-structural factor and well-being [27]. It describes the finding that as countries grow wealthier, average happiness levels do not increase accordingly. Later controlling for unobserved between-country differences, other authors found a clear income-happiness relationship [28–31]. Comparable to the well-being literature, a social gradient has been found for mental distress [12, 32, 33]. Rojas [34] summarizes theoretical approaches that strive to explain this relation: Absolute explanation approaches assume that income satisfies basic needs which enhances well-being, whereas relative explanations suppose that changing standards based on individual expectations and social comparisons alter the impact of income on person’s subjective well-being. An example for the latter are findings from the European Social Survey that found that reference income affects individual well-being negatively [35]. Following the relative explanation thesis, we expect that higher subjective evaluation of wealth is a negative predictor of mental distress.

#### Justice

The socioeconomic and macroeconomic context is also related to justice in the sense of fairness [36, 37]. As mentioned above, a given economic distribution might be evaluated as just by some and as unjust by others. Even though justice in general is a notion of the macrosystem, it relies on the individual’s judgment [38]. In turn, perceived justice has been shown to be related to clear health benefits [10]. Numerous studies found that the so-called *Belief in a Just World* is positively associated with mental health and negatively with mental distress (e.g., [39, 40]). To sum up, the perception of justice should be considered as a valuable link that might connect the macrosystem with individual mental distress. Higher perceived justice is assumed to be a protective factor in relation with mental distress because it provides a feeling of predictability, control and safety.

#### Freedom

In the context of political psychology, freedom has been proposed to complement justice as a second dimension of political values [41]. In their cross-cultural longitudinal study in 52 countries, Inglehart, Foa, Peterson, & Welzel [42] concluded that the perception of free choice increases happiness. However, freedom seems to be correlated with happiness only in rich countries and only if opportunity and capability coincide [43]. Twenge opposes this finding as he declares that too much freedom can lead to poor outcomes: people are paralyzed by their choices and then blame themselves when things go wrong. Hence, greater autonomy may lead to increased challenges and excitement, but also to greater isolation from others, and thus to higher levels of free-floating anxiety (p. 1017, [44]). All these findings suggest that freedom is a relevant characteristic of the macrosystem that seems to be related to mental distress, but the exact link is not yet clarified. Hence, we only expect a small correlation between freedom and mental distress because positive and negative effects might counterbalance each other.

Overall, wealth, justice and freedom can be conceptualized as characteristics of the respective macrosystem. In our study these macrosystems differ between countries. However, there might be a difference whether wealth, justice and freedom are evaluated for the country in general or for the personal situation within the country specifically. Therefore, we assessed both frames of reference in the present study.

### The present study

First, we know that macro-level factors are related to health and well-being. However, we know little about their relation to mental distress. Hence, symptoms of depression, anxiety and stress were the central dependent variables in the present exploratory study. Self-rated health was included for replication. Second, the study was carried out in eight countries with different welfare systems to reflect varying macro-systemic contexts. Third, instead of objective indicators that most often measure macro-level factors, we assessed subjective perceptions of macro-level factors in reference to the country in general and to the personal situation more specifically. All assessments were comparable across countries.

## Materials and method

### Participant characteristics

The population based survey comprised about 1000 participants per country (see Table 1 for a detailed sample description). Percentage of female participants varied significantly between 48% in Poland and 58% in Russia, χ^*2*^(7) = 20.68, *p* = .004. The average age ranged from 45 years in Poland to 58 years in the UK and differed significantly across countries, *F*(7) = 75.97, *p* < .001. The populations showed different distributions of marital status, χ^*2*^(21) = 171.77, *p* < .001, current work status, χ^*2*^(49) = 762.85, *p* < .001, and highest level of education, χ^*2*^(21) = 1281.3, *p* < .001.

**Table 1:** Sociodemographic variables of the participants.

		ES	FR	GE	PL	RU	SV	UK	US	Test-Statistic^2^
Participants	N	1006	1001	1001	1003	1010	1002	1002	1025	
Gender	% female	52	53	53	48	58	51	54	53	20.68(7)**
Age	*M* *(SD)*	48 (17)	51 (18)	52 (19)	45 (16)	45 (17)	56 (19)	58 (18)	51 (17)	75.97(7)***^3^
Marital status^1^										171.77(21)***
	Unmarried / Single	30	28	21	24	21	29	22	22	
	Married / Legal partnership	48	50	56	62	54	46	47	54	
	Widowed	9	12	13	6	12	11	17	9	
	Divorced / Separated	13	11	10	7	13	13	14	14	
Current main labor status^1^										762.85(49)***^4^
	Paid work	52	47	52	61	51	51	40	50	
	Education	3	3	7	7	2	3	2	3	
	Unemployed	14	7	3	6	7	2	3	5	
	Permanently sick or disabled	2	2	1	1	3	5	3	8	
	Retired	19	33	33	21	24	36	45	23	
	Community or military service	-	-	0	-	-	-	-	-	
	Housework, looking after children, other	6	5	3	4	10	1	4	6	
	Other	3	4	2	-	3	1	3	5	
Highest level of education^1^										1281.3(21)***
	10 years of school and below	36	15	7	22	3	17	20	6	
	High school graduation (12–13 years of school)	28	42	51	41	14	35	26	40	
	Vocational training, college graduation	18	24	21	10	36	26	35	35	
	Post-graduate / University degree	18	18	21	27	47	22	18	18	

*Note*. ES: Spain, FR: France, GE: Germany, PL: Poland, RU: Russia, SV: Sweden, UK: United Kingdom, US: United States of America;

*p ≤ .05;

** *p* ≤ .01;

*** *p*≤ .001

^1^Frequencies are presented in percent.

^2^Group differences are calculated with χ^*2*^-test or oneway ANOVA.

^3^Levene’s test indicated heterogeneity of variances *(F =* 5.17***)

^4^Expected values are in part below 5 in the contingency table, hence the result of the χ^2^-test might be incorrect.

### Measures

#### Sociodemographic variables

Gender, age, marital status, current main labor status and educational level were assessed according to the guidelines of the Task Force on Core Social Variables [45]. Educational level was harmonized following the International Standard Classification of Education [46, 47]. The harmonized educational data were categorized following Kraus et al. [48]: 0 (*10 years of school and below)*, 1 (*high school education (12–13 years of school)*, 2 (*vocational training*, *college graduation)*, 3 *(post-graduate*, *university degree)*.

#### Depression, anxiety and stress scales

Symptoms of depression, anxiety and stress were assessed with selected items of the Depression, Anxiety and Stress Scales (DASS-42) [49]. Three 7-item subscales for depressive, anxiety and stress symptoms over the past week were used. Items are rated on a 4-point Likert scale from 0 (*did not apply to me at all)* to 3 (*applied to me very much or most of the time)*. Cronbach’s alpha was good for the depression scale (α = .85), the anxiety scale (α = .80) and the stress scale (α = .85). In cross-cultural settings, the DASS has been validated and shown to be measurement invariant at least on a metric level [50–52]. This is a sufficient precondition for regression analysis. However, mean comparisons bias cannot be ruled out completely [53].

#### Self-rated health

Self-rated health was assessed as present-day health status on a scale from 0 to 100. 0 presented the worst imaginable health state and 100 presented the best imaginable health state.

#### Single-item questions on perceived wealth, justice and freedom

Single-item questions were used to assess perceived wealth, justice and freedom. To differentiate country characteristics from individual characteristics, we asked for perceived wealth, justice and freedom in reference to the country (called ‘country’s wealth/justice/freedom’ from hereon) and in reference to the personal situation (called ‘personal wealth/justice/freedom’ from hereon). All questions were phrased as follows: “When you compare [insert country name, e.g., the USA] to other countries, how wealthy do you find [insert country name, e.g., the USA]?” for country’s wealth and “When you compare yourself with other people in [insert country name, e.g., the USA], how wealthy do you feel?” for personal wealth; “When you compare [insert country name] to other countries, how fair do you find [insert country name]?” and “When you compare yourself with other people in [insert country name], how fairly do you feel treated?” for justice; “When you compare [insert country name] to other countries, how free do you find [insert country name]?” and “When you compare yourself with other people in [insert country name], how free do you feel?” for freedom. Responses could be given on a scale from 0 (*not at all wealthy*) to 100 (*very wealthy)*.

### Procedure

An independent social market and research institute conducted population-based surveys in Spain (ES), France (FR), Germany (GE), Poland (PL), Russia (RU), Sweden (SV), the United Kingdom (UK) and the United States of America (US). France and Germany represented conservative welfare states, Sweden exemplified a social welfare state, the UK and the US embodied liberal welfare states. Spain has mixed welfare state elements which is nowadays called “southern” welfare state and Poland and Russia are “post-soviet” welfare states [24].

The questionnaire was translated if translated versions of the respective set of questions were not available. Sociodemographic questions were extracted from the European Social Survey. The translations of the DASS-21 were downloaded from the DASS website in English, French, German, Polish, Spanish and Swedish [54]. A Russian version was translated based on the German version. However, instead of the DASS-21, the first 21 items of the DASS-42 were accidentally used for the French, German, Russian, Spanish and Swedish versions. As recommended by Lovibond & Lovibond [55], we adjusted the sum scores of the scales according to population-based correction factors to receive the best possible estimate of the DASS-21. All remaining questions were translated into the required languages following the procedure proposed by Wild and colleagues [56]. Language of reference was German as it is the native language of the research team.

From June to October 2014, participants were recruited from the residential populations aged 18 years and above via landline or mobile phone according to the dual-frame approach [57]. Regional, age and gender stratification was implemented to achieve representativeness. Kish selection grid was used to choose the person for the interview [58] and then computer-assisted telephone interviews were conducted. Explicit inclusion or exclusion criteria did not exist. The study was introduced and participants were asked for informed consent prior to each interview. In total, 8,027 interviews with a mean duration of 16 minutes were completed. Response rate estimates, based on the proportion of actual eligible cases out of cases of unknown eligibility [59], varied between 13.4% and 20.1%. The total response rate across all countries was 16.55%.

Informed consent was assessed verbally because the interview was conducted on the phone. If informed consent was not given, the interview was not continued. Agreement to the informed consent was coded in the raw data file. The Ethical Committee of the Faculty of Psychology at the Ruhr-Universität Bochum formally approved the study including the procedure to assess and document informed consent.

### Data analysis

Statistical analyses were conducted using SPSS version 21 [60] and R [61]. For analysis and graphical reporting in R, the packages car [62], lavaan [63], psych [64] and ggplot2 [65] were used.

Missing values for all variables included in the analysis are presented in Table 2. Across all countries, perceived country’s justice had the largest amount of missing values, whereas all other variables had missing values of less than 5%. Correlations and regressions were analyzed pairwise to consider missing values in the analyses. To avoid bias due to scaling, all values were z-standardized.

**Table 2:** Descriptive properties (mean, standard deviation skew, kurtosis, inter-item correlations Cronbach’s alpha) for all macro-level factors, mental distress and self-rated health.

Variable	*n*	*M*	*SD*	*Skew*	*Kurt*		Correlations												*α*	Missing
						gender	age	edu	wC	wP	jC	jP	fC	fP	health	dep	anx	stress		*%*
gender							.06	-.01	-.02	-.05	.00	.01	.04	.04	-.05	.04	.07	.07		0.00
age								-.12	.08	.05	.12	.10	.11	.11	-.27	.02	.01	-.11		0.53
education									.07	.10	.09	.12	.05	.03	.11	-.13	-.15	-.04		3.40
wealthC	7710	65.69	22.87	-0.52	-0.41					.46	.57	.46	.48	.38	.13	-.15	-.14	-.14		4.22
wealthP	7825	62.89	20.99	-0.35	-0.25						.34	.44	.33	.40	.24	-.23	-.18	-.20		2.80
justiceC	7588	62.73	23.16	-0.47	-0.42							.62	.56	.42	.13	-.15	-.14	-.15		5.74
justiceP	7796	69.37	21.88	-0.71	0.01								.50	.50	.19	-.22	-.20	-.20		3.16
freedomC	7761	77.28	21.02	-1.13	0.86									.66	.13	-.14	-.13	-.14		3.59
freedomP	7879	80.57	19.81	-1.29	1.50										.17	-.20	-.16	-.19		2.12
health	7684	76.57	20.13	-1.22	1.44											-.28	-.29	-.17		4.55
dep	7848	7.01	8.29	1.66	2.73												.71	.69	.85	2.51
anx	7874	6.14	7.44	1.65	2.78													.66	.80	2.19
stress	7885	11.29	9.14	0.78	0.06														.85	2.05

*Note*.

justiceC = Perceived justice in the country

justiceP = Perceived justice of the personal situation

freedomC = Perceived freedom in the country

freedomP = Perceived freedom of the personal situation

wealthC = Perceived wealth in the country

wealthP = Perceived wealth of the personal situation

As descriptive properties, mean, standard deviation, skewness, kurtosis, inter-item-correlations and Cronbach’s α were calculated for each country separately. Absolute values larger than 2 for skewness or larger than 7 for kurtosis were considered as reference for substantial non-normality as it is recommended for samples larger than 300 [66,67]. Cronbach’s alpha indicated internal reliability and was considered acceptable above α ≥ .70 [68].

Prior to mean comparisons of the Depression, Anxiety and Stress Scales as well as of the subjective macro-level factors across countries, the homogeneity of variances was analyzed with Levene’s test [69]. Variances turned out to be heterogeneous. Therefore, we used oneway-ANOVA based on Welch to compare means [70]. Squared Eta was used as effect size measure for oneway-ANOVA and Cohen’s *d* was calculated as effect size for post-hoc tests that were conducted as t-tests. According to Cohen [71], a small effect is *d* > .20, a medium effect is *d* > .50 and a large effect is indicated by *d* > .80.

Regression models were based on bivariate linear regression analysis. All country samples were combined to analyze the effect of subjective macro-level factors on depression, anxiety, stress and self-rated health across countries simultaneously. For that reason, seven dummy-variables based on a contrast matrix with mean differences between the US as reference country and all other countries were constructed to control for country differences within this regression model. This procedure enables a general conclusion about the relation between subjective macro-level factors and mental distress on the one hand and a more specific conclusion in regard to whether this relationship explains country differences, too.

## Results

### Mental distress

#### Descriptive properties of mental distress and self-rated health

A detailed overview of the descriptive properties of depression, anxiety and stress symptoms as well as self-rated health can be found in Table 2. In all countries, skew and kurtosis did not imply large deviations from normality. The distributions of depression, anxiety and stress levels were left skewed whereas self-rated health was generally right skewed. Correlation patterns emerged as expected: Depression, anxiety and stress were negatively correlated with all macro-social variables whereas self-rated health was positively correlated with all of these variables. Mental distress and self-rated health correlated negatively. Depression, anxiety and stress were highly correlated across all countries.

#### Mean comparisons of depression, anxiety and stress and self-rated health

**Fig 1:**
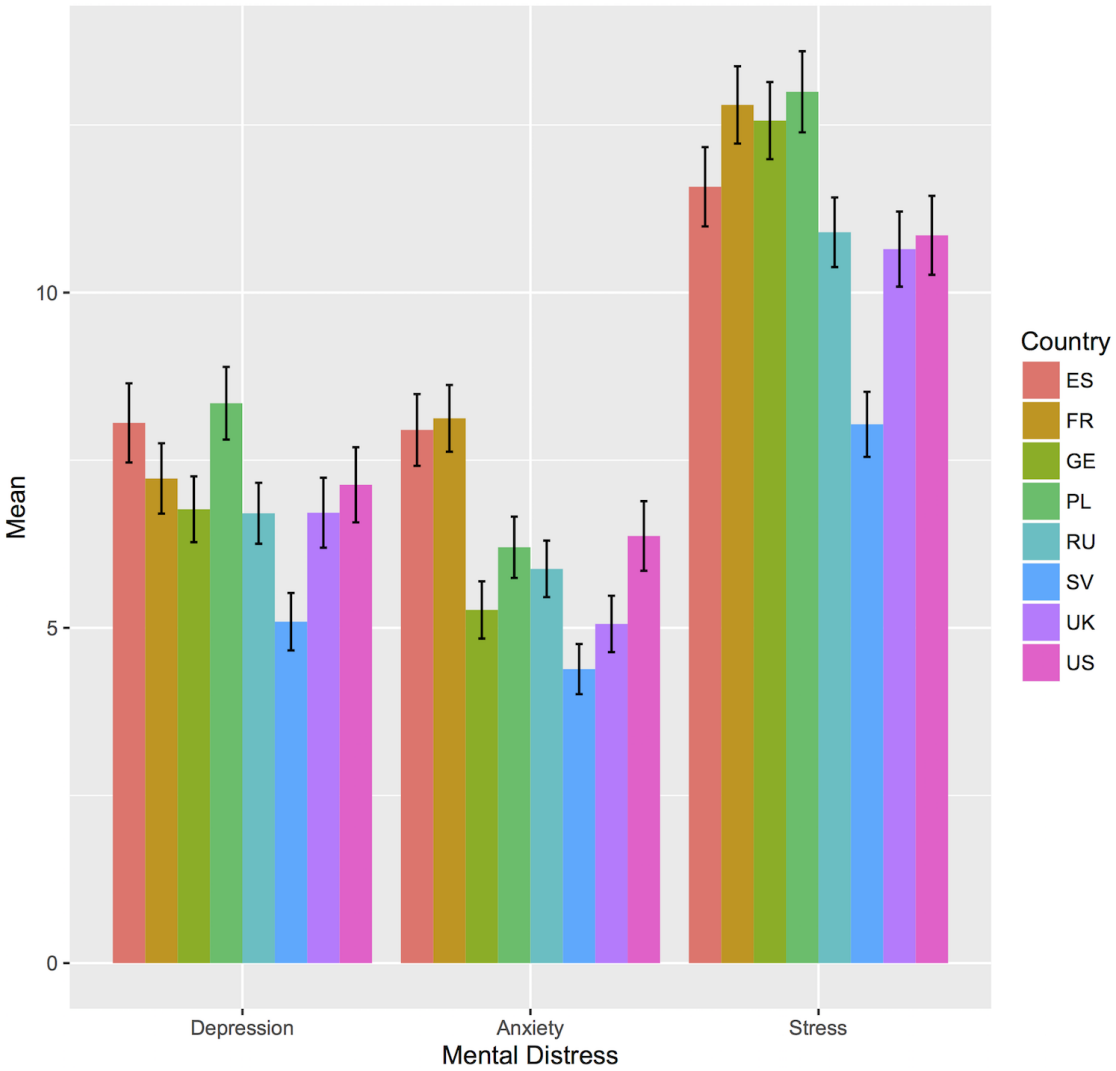
Mean differences of depression, anxiety and stress symptoms across countries. ES: Spain, FR: France, GE: Germany, PL: Poland, RU: Russia, SV: Sweden, UK: United Kingdom, US: United States of America.

**Fig 2:**
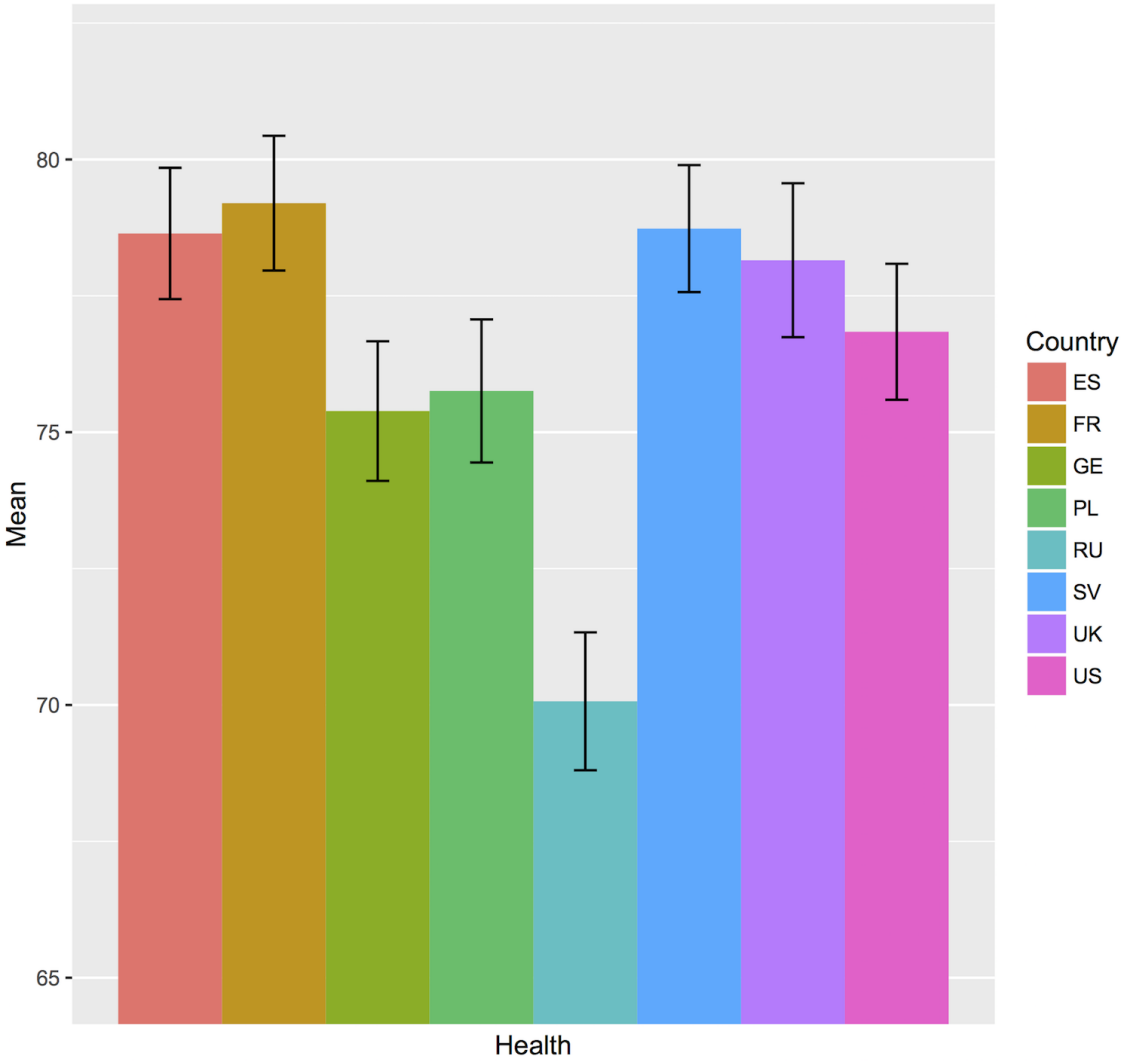
Mean differences of self-rated health across countries. ES: Spain, FR: France, GE: Germany, PL: Poland, RU: Russia, SV: Sweden, UK: United Kingdom, US: United States of America.

Levels of depression, *F*(7, 3356) = 16.26, *p* < .001, η^*2*^ = .01, anxiety, *F*(7, 3365) = 32.14, *p* < .001, η^*2*^ = .03, and stress symptoms, *F*(7, 3374) = 36.54, *p* < .001, η^*2*^ = .03, as well as self-rated health, *F*(7, 3276) = 21.92, *p* < .001, η^*2*^ = .02, differed significantly across all countries (Table 3). Post-hoc comparisons indicated that the lowest levels of mental distress were reported by Swedish participants for depression, anxiety and stress (see Fig 1). For depression, the symptom level in Sweden was significantly lower than all other countries’ ratings with small effects across country comparisons. The highest symptom levels were found in Spain and Poland. They differed significantly from Germany, Russia, Sweden, the UK and in part the US, however, effect sizes were negligible to small. The lowest anxiety ratings were reported by the German, Swedish and UK sample. In contrast, small to medium effects in post-hoc comparisons were found for the highest levels of anxiety in the Spanish and the French samples followed by the US sample. In general, stress levels were higher in comparison to depression and anxiety levels across all countries. Again, the Swedish sample reported significantly lower stress rates than all other samples with small to medium effects. The highest stress levels were indicated by the Polish sample closely followed by France and Germany. Small to medium effects were found for all three countries underlining significantly higher stress rates compared to Russia, Sweden, the UK and the US. Self-rated state of health was significantly higher in Spain, France and Sweden compared to Germany, Poland and Russia (see also Fig 2). The Russian sample’s self-rated health was significantly lower than all other countries’ health evaluations. The effect sizes for the comparisons to all other countries were low.

**Table 3:** Mean differences in health, depression, anxiety, and stress.

			*P*-values of post-hoc comparisons								Cohen’s *d* of post-hoc comparisons							
**Depression**				ES	FR	GE	PL	RU	SV	UK		ES	FR	GE	PL	RU	SV	UK
Levene test	*F*	12.00	FR	.76							FR	-						
	*df*	7.00	GE	.02	1.00						GE	.15	-					
	*p*	.00	PL	1.00	.07	.00					PL	-	-	.19				
Oneway ANOVA	*F*	16.26	RU	.01	1.00	1.00	.00				RU	.16	-	-	.21			
	*df*	7, 3357	SV	.00	.00	.00	.00	.00			SV	.36	.28	.23	.42	.23		
	*p*	.00	UK	.01	1.00	1.00	.00	1.00	.00		UK	.15	-	-	.19	-	.21	
	*η*^*2*^	.01	US	.36	1.00	1.00	.03	1.00	.00	1.00	US	-	-	-	.14	-	.25	-
**Anxiety**																		
Levene test	*F*	20.81	FR	1.00							FR	-						
	*df*	7.00	GE	.00	.00						GE	.35	.39					
	*p*	.00	PL	.00	.00	.13					PL	.22	.25	-				
Oneway ANOVA	*F*	32.14	RU	.00	.00	1.00	1.00				RU	.27	.31	-	-			
	*df*	7, 3365	SV	.00	.00	.21	.00	.00			SV	.49	.54	-	.27	.23		
	*p*	.00	UK	.00	.00	1.00	.01	.36	1.00		UK	.38	.42	-	.16	-	-	
	*η*^*2*^	.03	US	.00	.00	.02	1.00	1.00	.00	.00	US	.19	.22	.14	-	-	.27	.17
**Stress**																		
Levene test	*F*	13.07	FR	.08							FR	-						
	*df*	7.00	GE	.42	1.00						GE	-	-					
	*p*	.00	PL	.01	1.00	1.00					PL	.15	-	-				
Oneway ANOVA	*F*	36.54	RU	1.00	.00	.00	.00				RU	-	.22	.19	.23			
	*df*	7, 3374	SV	.00	.00	.00	.00	.00			SV	.41	.56	.53	.56	.36		
	*p*	.00	UK	.63	.00	.00	.00	1.00	.00		UK	-	.24	.21	.25	-	.31	
	*η*^*2*^	.03	US	1.00	.00	.00	.00	1.00	.00	1.00	US	-	.21	.18	.22	-	.32	-
**Health**																		
Levene test	*F*	4.15	FR	1.00							FR	-						
	*df*	7.00	GE	.01	.00						GE	.16	.19					
	*p*	.00	PL	.04	.00	1.00					PL	.14	.17	-				
Oneway ANOVA	*F*	21.92	RU	.00	.00	.00	.00				RU	.44	.46	.26	.28			
	*df*	7, 3276	SV	1.00	1.00	.01	.03	.00			SV	-	-	.17	.15	.45		
	*p*	.00	UK	1.00	1.00	.09	.30	.00	1.00		UK	-	-	-	-	.40	-	
	*η*^*2*^	.02	US	1.00	.25	1.00	1.00	.00	.98	.98	US	-	-	-	-	.34	-	-

*Note*. ES: Spain, FR: France, GE: Germany, PL: Poland, RU: Russia, SV: Sweden, UK: United Kingdom, US: United States of America

### Subjective macro-level factors

#### Descriptive properties of perceived wealth, justice and freedom

Table 2 also shows the descriptive properties of the subjective macro-level factors. Skew and kurtosis were within a normal range. Medium correlations were found between the evaluation of country’s and personal wealth, justice and freedom, respectively. Perceived country’s wealth also correlated to a medium extent with perceived country’s and personal justice and perceived country’s freedom. Similarly, perceived country’s justice and perceived country’s freedom showed a medium correlation as did perceived personal justice with both freedom ratings.

#### Mean comparisons of perceived wealth, justice and freedom

**Fig 3:**
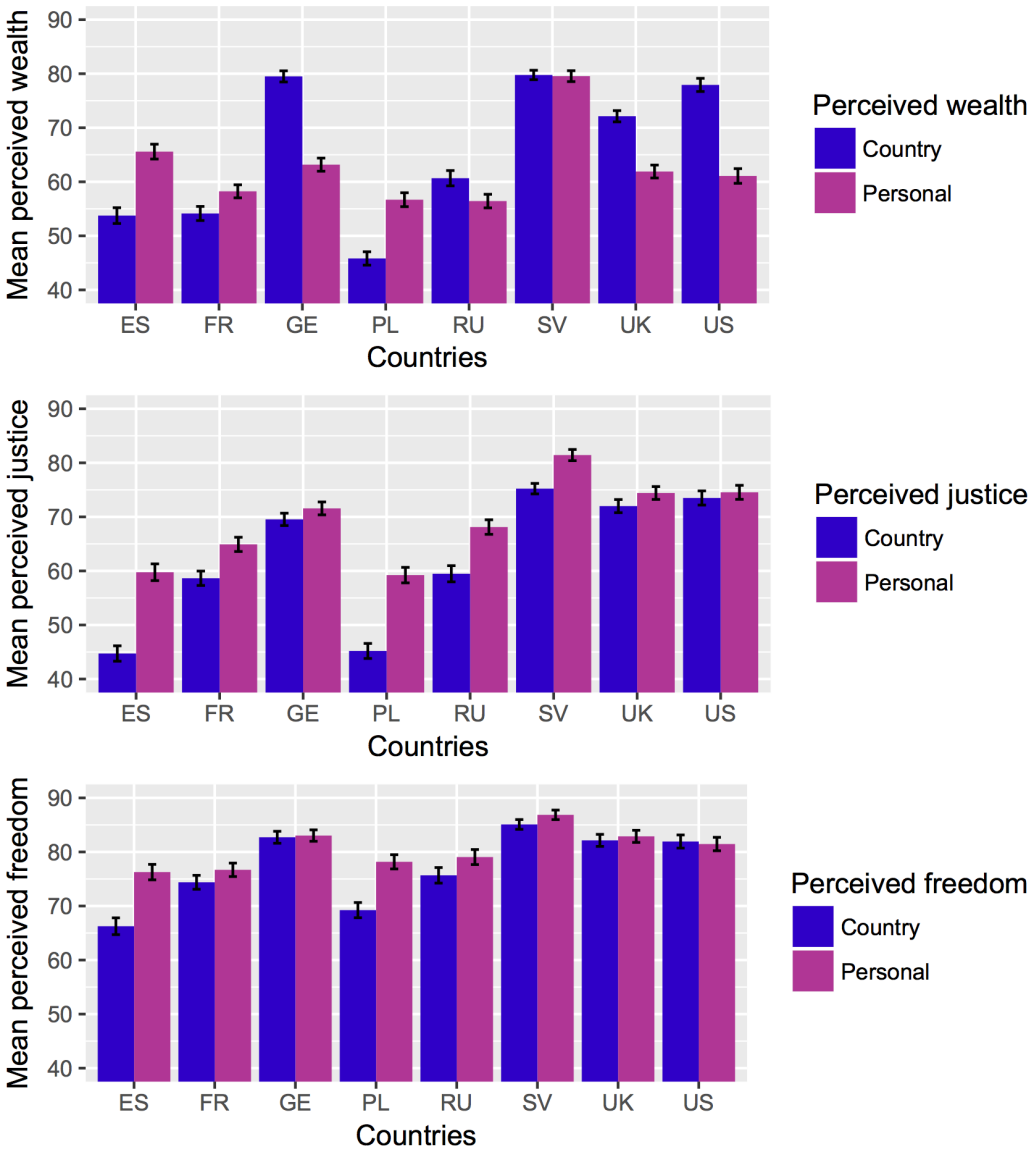
Mean differences of perceived wealth, justice and freedom across countries. ES: Spain, FR: France, GE: Germany, PL: Poland, RU: Russia, SV: Sweden, UK: United Kingdom, US: United States of America.

Mean comparisons of the subjective macro-level factors are shown in Fig 3. Each graph depicts the means of one subjective macro-level factor such as justice per country. Its evaluations referring to the situation in the country and to the personal situation are presented by bars in blue and pink.

Wealth is presented at the top of the graphic. Perceived country’s wealth, *F*(7, 3282) = 519.09, *p* < .001, η^*2*^ = .31, as well as perceived personal wealth, *F*(7, 3346) = 186.22, *p* < .001, η^2^ = .11, differ significantly across countries. Country’s wealth was rated lowest in Poland. Spain and France as well as Russia had slightly higher ratings than Poland, but the highest country’s wealth was perceived in the German and Swedish populations. Yet, only the Swedish population perceived its personal wealth equally high. Spanish, German, US and UK samples indicated similar rates of personal wealth that were all significantly lower than the Swedish evaluation. The lowest personal wealth ratings were found in the French, Polish and Russian samples.

The results for justice are shown in the middle graph. Again both ratings of perceived country’s justice, *F*(7, 3229) = 353.16, *p* < .001, η^*2*^ = .24 as well as perceived personal justice, *F*(7, 3329) = 144.70, *p* < .001, η^*2*^ = .11, differed significantly across all countries. The perceived country’s justice was rated lowest in Spain and Poland opposed by high ratings in Sweden, the UK and the US. The same pattern was found for perceived personal justice.

The bottom graphic depicts freedom that differed across countries, but effects were insignificant, *F*(7, 3307) = 112.32, *p* < .001, η^*2*^ = .09, in reference to the country and *F*(7, 3364) = 43.14, *p* < .001, η^*2*^ = .03, in reference to the personal situation. Country’s freedom was perceived highest in Sweden followed by Germany, the UK and the US, then Russia and finally Poland and Spain. Looking at perceived personal freedom, again the Swedish population rated its personal freedom highest, closely followed by German, UK and US ratings. The ratings of Spain, France, Poland and Russia were comparable and lower than the other countries.

### Predicting mental distress with subjective macro-level factors

Four regression models with symptoms of depression, anxiety and stress as well as self-rated health as outcome variables were tested controlling for country influences, gender, age and education. Perceived wealth, justice and freedom were subjective macro-level predictors. All regression models explained a small proportion of variance of depression, adjusted R^2^ = .08, anxiety, adjusted R^2^ = .08, and stress, adjusted R^2^ = .08, as well as self-rated health, adjusted R^2^ = .19 (Table 4).

**Table 4:** Predicting health, depression, anxiety and stress with the subjective evaluation of macro-social factors controlling for sociodemographic variables.

	Depression				Anxiety				Stress				Health			
	β	*se*	*t*	*p (>|t|)*	β	*se*	*t*	*p (>|t|)*	β	*se*	*t*	*p (>|t|)*	β	*se*	*t*	*p (>|t|)*
(Intercept)	-.03	.04	-0.93	.35	-.06	.04	-1.67	.10	-.14	.04	-3.73	.00	-.01	.03	-0.37	.71
Countries																
Spain	.09	.05	1.66	.10	.18	.05	3.53	.00	.05	.05	0.96	.34	.17	.05	3.44	.00
France	-.05	.05	-1.06	.29	.18	.05	3.74	.00	.16	.05	3.29	.00	.25	.05	5.52	.00
Germany	-.02	.04	-0.56	.57	-.12	.04	-2.73	.01	.22	.05	4.87	.00	-.01	.04	-0.34	.73
Poland	.06	.05	1.23	.22	-.09	.05	-1.77	.08	.12	.05	2.28	.02	.01	.05	0.19	.85
Russia	-.02	.05	-0.34	.73	.00	.05	-0.08	.94	-.04	.05	-0.80	.42	-.37	.05	-7.98	.00
Sweden	-.07	.05	-1.64	.10	-.13	.05	-2.83	.00	-.12	.05	-2.67	.01	.03	.04	0.66	.51
United Kingdom	-.07	.05	-1.56	.12	-.19	.05	-4.07	.00	.03	.05	0.71	.48	.26	.05	5.67	.00
Sociodemographic variables																
Gender	.06	.02	2.47	.01	.13	.02	5.58	.00	.14	.02	6.03	.00	-.04	.02	-2.05	.04
Age	.05	.01	4.17	.00	.02	.01	1.79	.07	-.08	.01	-6.60	.00	-.30	.01	-26.63	.00
Education	-.09	.01	-7.82	.00	-.11	.01	-9.45	.00	-.01	.01	-0.86	.39	.09	.01	8.01	.00
Macro-social factors																
Country																
Wealth	.00	.02	0.28	.78	.01	.02	0.51	.61	.00	.02	0.10	.92	-.01	.02	-0.50	.62
Justice	.02	.02	0.98	.33	.01	.02	0.73	.47	-.01	.02	-0.35	.73	.00	.02	0.22	.83
Freedom	.03	.02	1.82	.07	.01	.02	0.76	.45	.02	.02	1.19	.23	.00	.02	-0.04	.97
Personal																
Wealth	-.14	.02	-9.10	.00	-.08	.01	-5.61	.00	-.09	.02	-6.04	.00	.16	.01	10.81	.00
Justice	-.12	.02	-7.15	.00	-.12	.02	-7.34	.00	-.09	.02	-5.58	.00	.09	.02	6.00	.00
Freedom	-.10	.02	-5.79	.00	-.06	.02	-3.47	.00	-.09	.02	-5.27	.00	.11	.02	6.54	.00
Adjusted R^2^	.08				.08				.08				.19			

None of the subjective macro-level factors in relation to the country was a significant predictor of either of the outcome measures. In contrast, all subjective macro-level factors related to the personal situation were significant negative predictors of depression, anxiety and stress and positive predictors of self-rated health. Perceived personal wealth was the strongest predictor of symptoms of depression and self-rated health. The strongest predictor of anxiety was perceived personal justice. Perceived personal wealth, justice and freedom were equally strong predictors of stress.

All significant country differences were explained by gender, age, education and subjective macro-level factors for symptoms of depression. For self-rated health, anxiety and stress, some country differences were still significant and are hence related to variables that were not included in the model.

## Discussion

The aim of the present study was to explore the relation between symptoms of depression, anxiety and stress and perceived wealth, justice and freedom as subjectively evaluated macro-level factors. The study was carried out in eight countries representing different welfare systems in order to include a variety of macrosystems. To our knowledge, this is the first study to assess subjective evaluations of three macro-level factors comparably across eight countries’ populations. Results indicated that symptoms of depression, anxiety and stress as well as self-rated health differed across country populations and that subjective macro-level factors could partly explain those differences.

Mean comparisons of the DASS scales showed that the Swedish sample reported the lowest symptom levels of depression, anxiety and stress. If we take into account the welfare system as a macrostructural factor to interpret these results, a possible explanation for the good mental health of the Swedish sample could be the Scandinavian (social) welfare system which might systematically enforce characteristics that are associated with good health [4]: low inequality [12], social cohesion and integration [72]. This result corresponds to existing findings in regard to self-rated health that has been found to be higher in social welfare systems, too [4]. The subjective evaluations of wealth, justice and freedom in the present study support this thesis. The Swedish population reported high ratings for all macro-social variables in reference to the country as well as to their personal situation. This rating suits the findings of objective indicators where Sweden has the highest Gross Domestic Product [73], the lowest income inequality [74] and highest justice index [75] of the countries under study. Also, the Freedom Index, an objective rating of freedom, of Sweden is high in comparison to other countries, but not as high Spain, U.K. and U.S.A. in the present country sample [76].

In contrast, Spanish and Polish samples had the highest ratings of depressive symptoms, Spanish and French samples had the highest anxiety ratings, and the Polish sample, followed by French and German samples, had the highest ratings of stress. Self-rated health was lowest in Russia. From a historical and economic perspective, grave political and economic changes in recent years might explain these findings: In Eastern Europe, the transition of the Soviet Union to independent Post-Soviet countries and partly even European Union member states was accompanied by a mortality crisis associated with poorer health outcomes (e.g., [77–79]). In Southern European states, such as Spain in the present sample, but also Greece and Portugal, the economic crisis in the late 2000s is associated with increasing prevalence rates of mental disorders and higher suicide rates (e.g., [80–84]). These results correspond not only to higher symptom levels reported in Eastern and Southern European countries in the present study, but also to the lower ratings of subjective macro-level factors. Objective measures of macro-level factors reflect a low Gross Domestic Product in Poland, Russia and Spain [73], high income inequality and low objective ratings of justice in France, Poland, Russia and Spain [74,75] and low ratings of freedom in Poland and Russia [76].

The regression analysis demonstrated that subjective macro-level factors matter. Large effects were not expected for macro-level factors in relation with mental distress and self-rated health, since the macrosystem is at the utmost edge of the continuum of natural systems whereas the individual is usually positioned in the center [16]. Hence, a number of other factors are assumed to be more closely related to mental distress. Nevertheless, we found a small proportion of explained variance for our models and the adjusted R^2^ found in the present study are comparable to the explained variance in other studies investigating the macrosystem [4, 85]. Additional reasons for the small proportion of explained variance could be the single items that were used to assess the subjective macro-level factors and the relatively small variability of the countries’ macrosystems. Even though we had aimed at assessing a heterogenic sample of countries to ensure a large variability in the assessment of subjective macro-level factors, we had to rely on industrialized countries to reliably conduct the surveys. Hence, the macrosystems are still alike. In future studies, it would be interesting to extend and compare the present results to studies conducted in countries with completely different macrosystems such as the Chinese style of socialism, countries in transition such as Iraq or unstable countries like Democratic Republic of Congo. This might lead to a larger range of perceived wealth, justice and freedom which in turn could explain more variance in mental distress and self-rated health. Nevertheless, the present study is a first step to show that subjective macro-level factors predict mental distress and self-rated health to a small extend even in countries with rather similar macrosystems.

More specifically, we expected perceived wealth and justice to be negative predictors of mental distress whereas we only expected a small relationship between perceived freedom and mental distress, because we assumed positive and negative effects might counterbalance each other. As expected, perceived personal wealth, justice and freedom were significant negative predictors of depression and anxiety. Perceived personal freedom had the smallest predictive value. All three subjective macro-level factors predicted stress to the same extend and were positive predictors of self-rated health. Perceived wealth might be a secure and enriching foundation that prevents symptoms of depression and enhances health. This is in line with research that found a linear relationship between income and health when country differences were controlled for, which is also true for our study [28–31]. The perception of being treated justly might also prevent mental distress. It predicts symptoms of anxiety even more than wealth or freedom. This finding corresponds to the “status anxiety hypothesis” that postulates that inequality is linked to poorer health through the emerging sense of inferiority and associated negative feelings [86]. The feeling of being treated justly might take away the fear of sudden social decline and hence reduce anxiety. Finally, whereas differential predictive patterns are found for subjective macro-level factors in relation to depression, anxiety and self-rated health, our results indicate that the perception of good personal wealth, justice and freedom equally reduces the experience of stress.

Opposed to the predictive value of subjective macro-level factors related to the personal situation, perceived country’s wealth, justice and freedom were not significant predictors of depression, anxiety, stress and self-rated health. Similar results were found for the *Believe in a Just World*: The *personal* belief in a just world was more predictive for health outcomes than the *general* belief in a just world [39, 40]. Hence, in relation to mental distress, the relative personal evaluation seems to be more important than the evaluation of the absolute reference category (here the country).

### Limitations

The cross-sectional design that does not allow causal inferences limits the interpretation of our results. In the present study, we cannot rule out the possibility that the outlined effects may actually function in the other direction, in the sense that mental distress might shape the perception of wealth, justice and freedom, for example. Longitudinal or experimental designs are needed to ultimately clarify whether macro-level factors influence mental distress and health or whether the macrosystem is an outcome of population health.

Another limitation is the single-item design measuring subjective macro-level factors. We are aware that single-item questions are not ideal measures, especially for multi-faceted constructs such as justice and freedom that often have multiple, and sometimes incompatible meanings [87]. The absence of a measurement construct also limits the validity of the cross-country comparisons of the single-items because the differences due to variations in the translation, due to response or other biases cannot be excluded. Validated and longer questionnaires would have been a better choice to assess and compare these fundamental concepts across countries. However, the increasing number of surveys along with a widening range of long questionnaires have reduced potential participants’ motivation to take part in surveys [88]. Thus, brief instruments without redundant items are needed for social science and psychological research.

Another relevant aspect to consider is that the questions asked for comparisons in reference to other countries or other people in general. However, these reference categories were not specified further in our study. Therefore, we do not know if a participant chose his or her own family, neighbors or colleagues as reference category and whether this choice makes a difference. Happiness, for example, was systematically rated higher or lower depending on the references [89]. If reference categories varied across countries and participants, this might be a confounding factor that should be investigated in future studies.

The generalizability of the results needs to be considered with care, too. The total response rate across countries was approximately 17%, leading to a the nonresponse rate of 83%. Thus, there might be a nonresponse bias that should be kept in mind interpreting the data [90].

A final important point to consider is the personal sensitivity of an individual’s perception of subjective macro-level factors. Different sensitivities, as shown for justice sensitivity, for example, might lead to differential evaluations of perceived wealth, justice and freedom [36]. Similarly, mental distress might bias the evaluation of macro-level factors. However, the hypothesis that mental distress might bias the subjective evaluation of macro-level factors was experimentally investigated by Kraus et al. [48] who did not find that negative mood confounded the association between subjective SES and self-rated health.

### Perspectives and conclusion

Our findings underline the “invisible power of the macrosystem”. First of all, it is striking that the Swedish sample scores comparably low on symptoms of depression, anxiety and stress and high on self-rated health. One possible explanation could be that Sweden’s social welfare system creates a positive environment leading to lower mental distress and higher perceived health. The positive ratings of the subjective macro-level factors in the Swedish sample support this hypothesis. Second, perceived subjective macro-level factors in reference to the personal situation matter. Both findings stress the importance to consider the macrosystem as a social component of biopsychosocial models of mental distress. Assessing the subjective evaluation of macro-level factors might even explain how the utmost edge of the continuum of natural systems, the macrosystem, is actually related to the individual that is in the center of concentrically organized systems.

In her model of cumulative advantage and disadvantage, Thoits [91] sums up the damaging interaction between mental distress and inequality, such as gender or social class inequality. She warns that developments will proliferate if macro- and meso-level policy interventions do not address inequality. Taken further, the present study results suggest that not only inequality based on objective macro-level factors should be taken into account, but that the subjective experience—subjective macro-level factors—need to be considered, too. The additional subjective perspective could even help increase the precision of targeting and evaluating interventions [92].

It is clear that mental distress is related to macro-level factors and that taking these factors into account broadens the scope of the typically individual-centered therapy rationales. Hence, the WHO action plan for mental health is already pointing in the right direction including a macrosystemic focus to aim to reduce the burden of disease due to mental disorders [2].

## Supporting information

S1 File Questionnaire of the computer-assisted telephone interviews in English. (PDF)
