# Supplementary material for: Mental distress and perceived wealth, justice and freedom across eight countries: The invisible power of the macrosystem
Source: PLoS One. 2018 May 2;13(5):e0194642. doi: 10.1371/journal.pone.0194642 (PMC5931469; doi:10.1371/journal.pone.0194642)
Supplement: S1 File — (PDF) [file pone.0194642.s001.pdf]

Project: 140403_USA
Sprache: **englisch (USA)**
Datum: 05.11.2014

INT01

Good afternoon, my name is XX from USUMA GmbH, from an independent social research institute in Berlin.

We are doing a study about justice and well-being on behalf of Ruhr University Bochum.
A representative selection of citizens age 18 and over will be asked, where individuals will be determined according to an exclusive selection principle. Your household was selected as well. In order to get a meaningful result, the participation of a person in your household is very important.

First I would like to identify the person with whom we want to fill in the questionnaire in your household.
First, I need a few details of the persons in your household.

The conversation would not take longer than 12 minutes.

In the context of our scientific study we are interested in different aspects of personal well-being in the population and your personal estimate, for example, of justice and freedom in USA.

Cordially, we would ask you to support us. The participation is, of course, free. There will be no disadvantages if you do not participate at the study or those will be cancelled in the process. Your participation, however, will support our research.

Do you agree upon to start with the questionnaire?

SW1

Would you be so kind to tell me how many people live in your household who are 18 years and older, yourself included?

==> /Q100MOBIL=01

$E 1 9

Choices			
refused	99	N	==> SELS

In which state do you live?

Choices	
Alabama	01
Alaska	02
Arizona	03
Arkansas	04
California	05
Colorado	06
Connecticut	07
Delaware	08
District of Columbia	09
Florida	10
Georgia	11
Hawaii	12
Idaho	13
Illinois	14
Indiana	15
Iowa	16
Kansas	17
Kentucky	18
Louisiana	19
Maine	20
Maryland	21
Massachusetts	22
Michigan	23
Minnesota	24
Mississippi	25
Missouri	26
Montana	27
Nebraska	28
Nevada	29
New Hampshire	30
New Jersey	31
New Mexico	32
New York	33
North Carolina	34
North Dakota	35
Ohio	36
Oklahoma	37
Oregon	38
Pennsylvania	39
Rhode Island	40
South Carolina	41
South Dakota	42
Tennessee	43
Texas	44
Utah	45
Vermont	46
Virginia	47
Washington	48
West Virginia	49
Wisconsin	50
Wyoming	51

Q100

INT.: Please enter without asking!

Code sex

Choices	
male	01
female	02

Q101

Comment: Age only query directly when no age is still displayed.

Tell me first, how old are you?

$E 18 99

SA2A

Reference: Please click on the month.

First, I have a few questions for statistical purposes.
Please tell me your birth month?

Choices	
January	01
February	02
March	03
April	04
May	05
June	06
July	07
August	08
September	09
October	10
November	11
December	12
refused	99

SA2B

Please enter the 4-digit year. For example, 1961.

In what year were you born?

$E 1900 1996

Choices	
refused	KA

SA3US

comment: read out. Only one response.

Were you born in the US?

Choices	
yes	01
no	02
refused	99

SA3AUS

In which state were you born?

Choices	
Alabama	01
Alaska	02
Arizona	03
Arkansas	04
California	05
Colorado	06
Connecticut	07
Delaware	08
District of Columbia	09
Florida	10
Georgia	11
Hawaii	12
Idaho	13
Illinois	14
Indiana	15
Iowa	16
Kansas	17
Kentucky	18
Louisiana	19
Maine	20
Maryland	21
Massachusetts	22
Michigan	23
Minnesota	24
Mississippi	25
Missouri	26
Montana	27
Nebraska	28
Nevada	29
New Hampshire	30
New Jersey	31
New Mexico	32
New York	33
North Carolina	34
North Dakota	35
Ohio	36
Oklahoma	37
Oregon	38
Pennsylvania	39
Rhode Island	40
South Carolina	41
South Dakota	42
Tennessee	43
Texas	44
Utah	45
Vermont	46
Virginia	47
Washington	48
West Virginia	49
Wisconsin	50
Wyoming	51
other country	52
refused	99

SA5

comment: read out. Only one response.

Are you citizen of the US?

Choices		
yes	01	
no	02	
refused	99	

SA5A

Please specify your ethnicity.

Choices		
Hispanic or Latino	01	
Not Hispanic or Latino	02	
refused	99	

SA5B

Please specify your race.

Choices		
American Indian or Alaska Native	01	
Asian	02	
Black or African American	03	
Native Hawaiian or Other Pacific Islander	04	
White	05	
	06	
refused	99	X

SA6

Comment: note answer.

What language do you speak most often at home?

Choices		
arabic	01	
bulgarian	02	
chinese	03	
danish	04	
german	05	
english	06	
finnish	07	
french	08	
italian	09	
japanese	10	
korean	11	
kurdish	12	
dutch	13	
norwegian	14	
polish	15	
portuguese	16	
russian	17	
swedish	18	
spanish	19	
turkish	20	
Other, which ?	21	O
refused	99	

	Q1
Comment: Enter the number in the box below.	

To help people say how good or bad a health state is, please use the same scale on which the best state you can imagine is marked 100 and the worst state you can imagine is marked 0.
We would like you to indicate on this scale how good or bad your own health state is today, in your opinion.

$E 0 100

Choices	
refused	KA

	Q2
Comment: Enter the number in the box below.	

How do you see yourself: are you generally a person who is fully prepared to take risks or do you try to avoid taking risks?

Please imagine a scale, where 0 means "not at all willing to take risks",
100 means "very willing to take risks",
and the numbers in between mean intermediate degrees of willingness to take risks.

Please tell me the appropriate rating of willingness to take risks.

$E 0 100

Choices	
refused	KA

	Q3
Comment: Enter the number in the box below.	

This question deals with your appearance in comparison to other people.

How good or bad in your opinion is your appearance in comparison to the appearance of other people?

Please imagine a scale, where absolutely beautiful looking people are marked with "100" and absolutely ugly looking with "0".

Please tell me the appropriate rating of your appearance.

$E 0 100

Choices	
refused	KA

Darstellung als Schirm mit jeweiligem Fragenpaar

Q4A
Comment: In each case only one response.
Now we will talk about fairness, freedom, wealth and social connectedness.<br>We are interested in knowing how you evaluate USA. Please answer following questions in the respective area.<br><br>*The first area is fairness.*<br><br>When you compare the USA with other countries, how fair do you find the USA?<br>Please imagine a scale from 0 to 100. In the context this means ...<br>0 = not at all fair<br>100 = very fair<br>$E 0 100

Choices	
refused	999

Q5A
When you compare yourself with other people in the USA, how fairly do you feel treated?<br><br>Please imagine a scale from 0 to 100. In the context this means …<br>0 = not at all fair<br>100 = very fair<br>$E 0 100

Choices	
refused	999

Q6A
Comment: In each case only one response.
The second area is freedom.<br><br>When you compare the USA with other countries, how free do you find the USA?<br><br>Please remain at the scale from 0 to 100. Now this means…<br>0 = not at all free<br>100 = very free<br>$E 0 100

Choices	
refused	999

Q7A
When you compare yourself with other people in the USA, how free do you feel?<br><br>Please remain at the scale from 0 to 100. Now this means ...<br>0 = not at all free<br>100 = very free<br>$E 0 100

Choices	
refused	999

Q8A

Comment: In each case only one response.

The third area is wealth.

When you compare the USA with other countries, how wealthy do you find the USA?

Please remain at the scale from 0 to 100. Now this means ...
0 = not at all wealthy and
100 = very wealthy.
$E 0 100

Choices	
refused	999

Q9A

When you compare yourself with other people in the USA, how wealthy do you feel?

Please remain on the scale from 0 to 100. Now this means ...
0 = not at all wealthy
100 = very wealthy
$E 0 100

Choices	
refused	999

Q10A

Comment: In each case only one response.

The fourth area is the social connectedness of people with each other.

When you compare the USA with other countries, how connected are the people with each other in your opinion?

Please imagine a scale from 0 to 100. In the context this means ...
0 = not at all connected and
100 = very connected.
$E 0 100

Choices	
refused	999

Q11A

When you compare yourself with other people in the USA, how connected do you feel with other people?

Please imagine a scale from 0 to 100. In the context this means ...
0 = not at all connected and
100 = very connected.
$E 0 100

Choices	
refused	999

Q12A

Comment: Only one response. Please read the scale and the key points of the scale regularly.

Let´s deal now with stress and burden:

Please tell me at each statement how much these apply to you during the last week.
There are no right or wrong answers. Please try to decide spontaneously for an answer.

The scale, which is available to you, reaches from 0 to 3. In the context this means…
0 = never: did not apply to me at all
1 = sometimes: applied to me to some degree, or some of the time
2 = often: applied to me to considerable degree, or a good part of time.
3 = almost always: applied to me very much, or most of the time.

	0 = never: did not apply to me at all	1 = sometimes: applied to me to some degree, or some of the time	2 = often: applied to me to considerable degree, or a good part of time.	3 = almost always: applied to me very much, or most of the time.	refused
I found it hard to wind down.	☐	☐	☐	☐	☐
I was aware of dryness of my mouth.	☐	☐	☐	☐	☐
I couldn't seem to experience any positive feeling at all.	☐	☐	☐	☐	☐
I experienced breathing difficulty (e.g., excessively rapid breathing, breathlessness in the absence of physical exertion).	☐	☐	☐	☐	☐
I found it difficult to work up the initiative to do things.	☐	☐	☐	☐	☐
I tended to over-react to situations.	☐	☐	☐	☐	☐
I experienced trembling(e.g., in the hands).	☐	☐	☐	☐	☐
I found it difficult to relax.	☐	☐	☐	☐	☐
I was worried about situations in which I might panic and make a fool of myself.	☐	☐	☐	☐	☐
I felt that I had nothing to look forward to.	☐	☐	☐	☐	☐
I found myself getting agitated.	☐	☐	☐	☐	☐
I felt that I was using a lot of nervous energy.	☐	☐	☐	☐	☐
I felt down-hearted and blue.	☐	☐	☐	☐	☐
I was intolerant of anything that kept me from getting on with what I was doing.	☐	☐	☐	☐	☐
I felt I was close to panic.	☐	☐	☐	☐	☐
I was unable to become enthusiastic about anything.	☐	☐	☐	☐	☐
I felt I wasn´t worth much as a person.	☐	☐	☐	☐	☐
I felt that I was rather touchy.	☐	☐	☐	☐	☐
I was aware of the action of my heart in the absence of physical exertion. (e.g., sense of heart rate increase, heart missing a beat).	☐	☐	☐	☐	☐
I felt scared without any good reason.	☐	☐	☐	☐	☐
I felt that life was meaningless.	☐	☐	☐	☐	☐

Statistik 2. Teil

SB1UK
comment: read out. Only one response.
You were born in ...

Choices	
A big city	01
The suburbs or outskirts of a big city	02
A town or a small city	03
A country village	04
A farm or home in the countryside	05
refused	99

SB2UK
comment: read out. Only one response.
Which phrase describes best the area where you live?

Choices	
A big city	01
The suburbs or outskirts of a big city	02
A town or a small city	03
A country village	04
A farm or home in the countryside	05
refused	99

SB3US
What is your marital status?

Choices	
Now married	01
Widowed	02
Divorced	03
Separated	04
Never married	05
refused	99

SB4
comment: read out. Only one response.
Do you have children?

Choices	
No	01
Yes, 1 child	02
Yes, 2 children	03
Yes, 3 children	04
Yes, more than 3 children	05
refused	99

TEXT1

==> *IF((SB4=01),01,02)

Choices	
Do you want to have a child/children in the future?	01
Do you want to have a child/ more children in the future?	02

SB5

comment: Only one response.

<TEXT1>

==> /+1(Q100==2 AND Q101>45) OR (Q100==1 AND Q101>60)

Choices	
Yes	01
No	02
refused	99

SB6

Comment: Enter the number below.

Including yourself, how many people - including children - live here regularly as members of this household?

$E 1 15

Choices		
refused	99	N

SB7A

$R.1 0.5 110

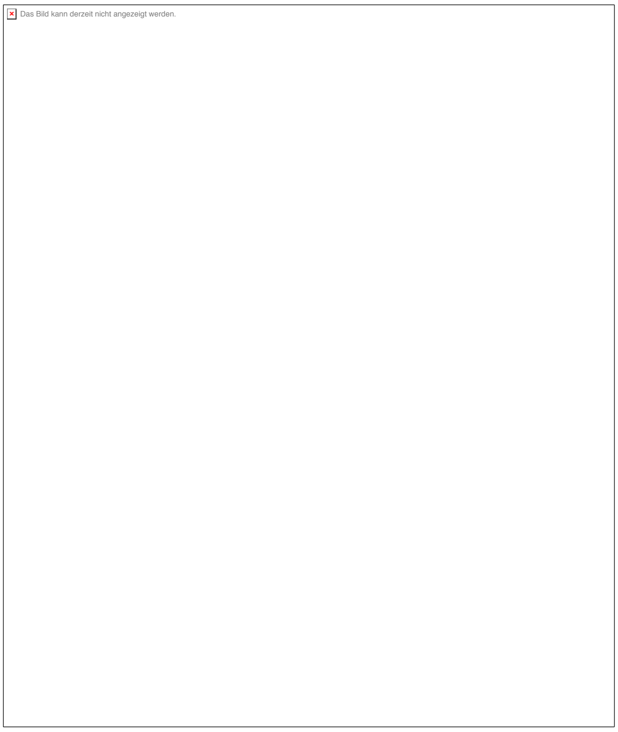

SB8

comment: read out. Only one response.

To which group do you belong ?

Choices	
A one-person household	01
More-people household	02
Single parent with children younger than 25 years	03
Parents with children younger than 25 years	04
Parents or single parent with children younger than 25 years or other people in the household.	05
Other household	06
refused	99

SB9US

Comment: Specified in Dollar. Enter in the box below Please .

Please tell me your household's total income, after tax and compulsory deductions, from all sources?
If you don't know the exact figure, please give an estimate.
Use what you know best: weekly, monthly or annual income.

Choices	
Less than $10,000	01
$10,000 to $19,999	02
$20,000 to $29,999	03
$30,000 to $39,999	04
$40,000 to $49,999	05
$50,000 to $59,999	06
$60,000 to $69,999	07
$70,000 to $79,999	08
$80,000 to $89,999	09
$90,000 to $99,999	10
$100,000 to $149,999	11
$150,000 or more	12
refused	99

SB10UK

comment: read out. Only one response.

Which of these descriptions applies to what you have been doing for the last 7 days?

Choices	
in paid work (or away temporarily) (employee, self-employed, working for your family business)	01
in education, (not paid for by employer) even if on vacation	02
unemployed and actively looking for a job	03
unemployed, wanting a job but not actively looking for a job	04
permanently sick or disabled	05
retired	06
doing housework, looking after children or other persons	07
other	08
refused	99

SB10AUS

Are you currently...?

Choices	
Employed for wages	01
Self-employed	02
Out of work and looking for work	03
Out of work but not currently looking for work	04
A homemaker	05
A student	06
Retired	07
Unable to work	08
refused	99

SB10BUS

Please describe your work.

==> +1NOT SB10AUS=01,02,05

Choices	
Employee of a for-profit company or business or of an individual, for wages, salary, or commissions	01
Employee of a not-for-profit, tax-exempt, or charitable organization	02
Local government employee (city, county, etc.)	03
State government employee	04
Federal government employee	05
Self-employed in own not-incorporated business, professional practice, or farm	06
Self-employed in own incorporated business, professional practice, or farm	07
Working without pay in family business or farm	08
refused	99

		SB11
comment: read out. Only one response.		
In your main job are/were you		
==> +1NOT SB10UK=01		

Choices		
an employee	01	
self-employed	02	
or, wokring for your own family's business	03	
refused	99	

		SB11AUK
Do you have a work contract of ...		
==> +1NOT (NATIO=001 AND SB10UK=01,02)		

Choices		
unlimited duration	01	
limited duration	02	
or, do you have no contract?	03	
refused	99	

		SB13US
comment: read out. Only one response.		
What is the highest degree or level of school you have completed?		

Choices		
No schooling completed	00	
Nursery school	01	
Kindergarten	02	
Grade 1 through 11, specify grade	03	O
12th grade - NO DIPLOMA	04	
Regular high school diploma	05	
GED or alternative credential	06	
Some college credit, but less than 1 year if college credit	07	
1 or more years of college credit, no degree	08	
Associate's degree (for example AA, AS)	09	
Bachelor's degree (for example: BA, BS)	10	
Master's degree (for example: MA, MS, Meng, MEDd, MSW, MBA)	11	
Professional degree beyond a bachelor's degree (for example: MD, DDS, DVM, LLB, JD)	12	
Doctorate degree (for example PhD, EdD)	13	
refused	99	

Darstellung als Schirm

SB14UK
Comment: Specified in inch, feet.
feet
$E 0 10

SB14AUK
inch
$E 0 11

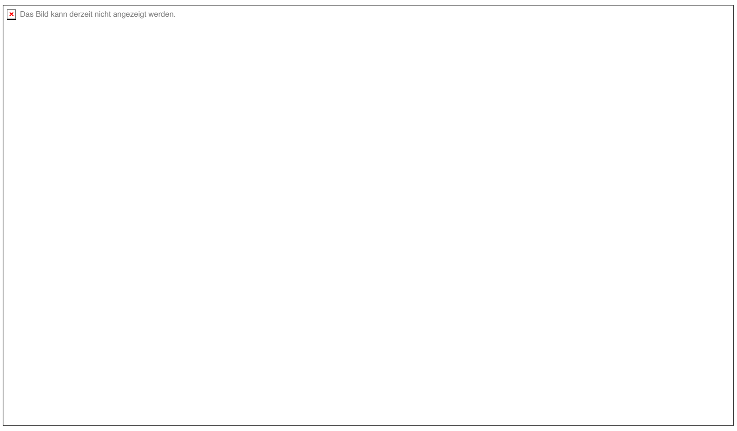

SB15US
Comment: Specified in lbs.
Please tell me your body weight:
$E 50 400

Choices	
refused	KA

SHM1
Do you use the cellular phone which I just called, exclusively only, do you share it with others or do you just use it by chance ?
==> /+3NOT MOBIL=01

Choices		
use cell phone alone	01	==> /+3
share it with others	02	
use it just by chance	98	==> /+3
refused	99	==> /+3

SHM1A

How many people from 14 years are there ?

Comment: Personal number without the questioning person.

==> /+1NOT SHM1=02

$E 1 20

Choices	
no further	00
refused	99

SHM2

Do you have at home a landline phone?
This means a phone by which you are accessible under your local prefix ?

Choices	
yes	01
no	02
refused	99

SHM3

About how many cellular phone numbers are you currently personally accessible by phone, in other words you use it to call, no matter whether professionally or private ?

Choices	
no (online landline)	00
one	01
two	02
three	03
four	04
five	05
refused	99

SHM4

Let´s get to your landline phone.
About how many different landline phone numbers with your prefix are you accessible at home ?

Thus we mean no calling numbers which exclusively belong to a fax machine or computer.

Choices	
no (online mobile)	00
one	01
two	02
three	03
four	04
five	05
d.k. / refused	99

				INT98
Sorry but you don't belong to our target group.<br>Thank you very much for your time! Good bye!				

Choices			
Screeninginterview	SC	D	==> /END

				INT99
Thank you very much for the interview!				

Choices		
Interview completet	VO	D

				BYEND
I wish you a nice day. Goodbye!				

Choices			
Interview completet	01	D	==> /QSEX

Stichprobenmerkmale

	NATIO
==> /+11>0	

Choices	
Deutschland	049
England	044
Frankreich	033
Spanien	034
Polen	048
Schweden	046
Russland	007
USA	001

	MOBIL

Choices	
Festnetz	00
Handy	01
